# Supplementary material for: Repeatability of evolution on epistatic landscapes
Source: Sci Rep. 2015 May 5;5:9607. doi: 10.1038/srep09607 (PMC5386189; doi:10.1038/srep09607)
Supplement: Supplementary Information [file srep09607-s1.pdf]

Supporting Information: Repeatability of evolution on epistatic landscapes

Benedikt Bauer* and Chaitanya S. Gokhale†
*Department of Evolutionary Theory,
Max Planck Institute for Evolutionary Biology,
August-Thienemann-Straße 2, 24306 Plön, Germany
†New Zealand Institute for Advanced Study,
Massey University, Auckland, New Zealand

General Probability Generating Functions

In the main text we considered only the case where each individual has to die or divide in every time step. Here we relax this assumption and consider a more realistic scenario where only some individuals proliferate or die, whereas others do not take any action at all (Fig. A.1). Then, the probability generating functions for the four types: wild type, individuals with mutation A, individuals with mutation B, and individuals with both mutations are defined as

$$\begin{aligned}
f_{ab}(s_{ab}, s_{Ab}, s_{aB}, s_{AB}) &= d_{ab} + (1 - b_{ab} - d_{ab})s_{ab} + b_{ab}((1 - \mu_A - \mu_B)s_{ab} + \mu_A s_{Ab} + \mu_B s_{aB})^2, \\
f_{Ab}(s_{ab}, s_{Ab}, s_{aB}, s_{AB}) &= d_{Ab} + (1 - b_{Ab} - d_{Ab})s_{Ab} + b_{Ab}((1 - \mu_B^A)s_{Ab} + \mu_B^A s_{AB})^2, \\
f_{aB}(s_{ab}, s_{Ab}, s_{aB}, s_{AB}) &= d_{aB} + (1 - b_{aB} - d_{aB})s_{aB} + b_{aB}((1 - \mu_A^B)s_{aB} + \mu_A^B s_{AB})^2, \\
f_{AB}(s_{ab}, s_{Ab}, s_{aB}, s_{AB}) &= d_{AB} + (1 - b_{AB} - d_{AB})s_{AB} + b_{AB}s_{AB}^2. \qquad \text{(A.1)}
\end{aligned}$$

The functions are similar to the scenario of binary splitting (cf. Eq. 8 in the main text). There is only one term added: $(1 - b_i - d_i)s_i, i \in \{ab, Ab, aB, AB\}$ which denotes the case of the individual neither dividing nor dying. To make the model even more realistic one could also

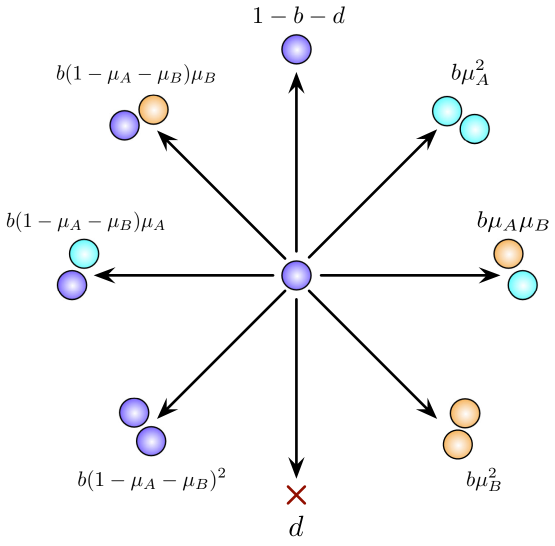

Figure A.1: **Process described by the general pgf.** An individual can either die, proliferate, or neither and just live. If it proliferates the offspring can mutate. In case of including back mutations additional mutation terms appear leading as in Eq. (A.2).

include back mutations,

$$\begin{aligned}
f_{ab}(s_{ab}, s_{Ab}, s_{aB}, s_{AB}) &= d_{ab} + (1 - b_{ab} - d_{ab})s_{ab} + b_{ab}((1 - \mu_A - \mu_B)s_{ab} + \mu_A s_{Ab} + \mu_B s_{aB})^2 \\
f_{Ab}(s_{ab}, s_{Ab}, s_{aB}, s_{AB}) &= d_{Ab} + (1 - b_{Ab} - d_{Ab})s_{Ab} + b_{Ab}((1 - \mu_{ab}^A - \mu_B^A)s_{Ab} + \mu_{ab}^A s_{ab} + \mu_B^A s_{AB})^2 \\
f_{aB}(s_{ab}, s_{Ab}, s_{aB}, s_{AB}) &= d_{aB} + (1 - b_{aB} - d_{aB})s_{aB} + b_{aB}((1 - \mu_{ab}^B - \mu_A^B)s_{aB} + \mu_{ab}^B s_{ab} + \mu_A^B s_{AB})^2 \\
f_{AB}(s_{ab}, s_{Ab}, s_{aB}, s_{AB}) &= d_{AB} + (1 - b_{AB} - d_{AB})s_{AB} \\
&\quad + b_{AB}\left((1 - \mu_A^{AB} - \mu_B^{AB})s_{AB} + \mu_A^{AB} s_{Ab} + \mu_B^{AB} s_{aB}\right)^2
\end{aligned} \tag{A.2}$$

If the fitness landscape is rugged, i.e. having multiple local optima, they would be inaccessible from certain “downstream” directions if back mutations are not allowed. Hence allowing back mutations, allows to have a rugged fitness landscape with local optima accessible from multiple directions. The probability generating functions seem more complex, but the principle of the computation as discussed in the main text does not change at all.

Time distribution

Here, we give a more detailed description on how to calculate the time distribution for the minimal model with four types, and two paths, but with back mutations.

1. Calculate the extinction probability of the final mutant type AB as in [1]

$$e_{AB} = \frac{d_{AB} + b_{AB}\left(\mu_A^{AB} + \mu_B^{AB}\right)^2}{b_{AB}(1-\mu_A^{AB} - \mu_B^{AB})^2}. \tag{A.3}$$

   Note, that without back mutations the extinction probability reduces to $e_{AB} = \frac{d_{AB}}{b_{AB}}$ as in the main text.

2. Until some t_{max} calculate recursively

$$\begin{aligned}
f_{AB}^{\circ(t)} &= d_{AB} + (1 - b_{AB} - d_{AB}) f_{AB}^{\circ(t-1)} \\
&\quad + b_{AB}\left((1-\mu_A^{AB} - \mu_B^{AB}) f_{AB}^{\circ(t-1)} + \mu_A^{AB} f_{Ab}^{\circ(t-1)} + \mu_B^{AB} f_{aB}^{\circ(t-1)}\right)^2, \\
f_{aB}^{\circ(t)} &= d_{aB} + (1 - b_{aB} - d_{aB}) f_{aB}^{\circ(t-1)} + b_{aB}\left((1-\mu_A^{B} - \mu_{aB}^{B}) f_{aB}^{\circ(t-1)} + \mu_{aB}^{B} f_{ab}^{\circ(t-1)} + \mu_A^{B} f_{AB}^{\circ(t-1)}\right)^2, \\
f_{Ab}^{\circ(t)} &= d_{Ab} + (1 - b_{Ab} - d_{Ab}) f_{Ab}^{\circ(t-1)} + b_{Ab}\left((1-\mu_B^{A} - \mu_{aB}^{A}) f_{Ab}^{\circ(t-1)} + \mu_{aB}^{A} f_{ab}^{\circ(t-1)} + \mu_B^{A} f_{AB}^{\circ(t-1)}\right)^2,
\end{aligned} \tag{A.4}$$

$$\begin{aligned}
f(t) := f_{ab}^{\circ(t)} &= d_{ab} + (1 - b_{ab} - d_{ab}) f_{ab}^{\circ(t-1)} \\
&\quad + b_{ab}\left((1-\mu_A - \mu_B) f_{ab}^{\circ(t-1)} + \mu_A f_{Ab}^{\circ(t-1)} + \mu_B f_{aB}^{\circ(t-1)}\right)^2
\end{aligned}$$

   where $f_{aB}^{\circ(0)} = f_{Ab}^{\circ(0)} = f_{ab}^{\circ(0)} = 1$ and $f_{AB}^{\circ(0)} = e_{AB}$. Note, that without back mutations these functions would not be coupled anymore and one can first calculate f_{Ab}^t and f_{aB}^t for all t, since those functions would not depend on f_{ab}. Moreover, $f_{AB}^{\circ(t)}$ would be equal to $e_{AB}\ \forall\ t$. Hence, one would not need to recursively calculate $f_{AB}^{\circ(t)}$. However, the complexity does not change.

3. The probability to get the final, successful AB mutant, i.e. an individual that produces a lineage that does not die out again, exactly at time t is

$$\tau(t) = f^N(t-1) - f^N(t). \tag{A.5}$$

   where N is the number of individuals in the beginning. Calculating this for all $t \in \{0, \ldots, t_{max}\}$ we obtain the time distribution.

Single-Path time distribution

Here, we explain the computation of the probability distribution of the pathway via type Ab exemplarily. Allowing back mutations it is unclear how to specify different mutational pathways. For instance for the pathway $ab \to aB \to ab \to Ab \to AB$ it is obscure to say via which type the final mutant has been reached. Obviously the final mutant has been reached via type Ab, but it might be necessary for the population to first reach type aB. Hence, aB might play a vital role for reaching AB, too. For this reason we neglect back mutations in the computation of the path probabilities, thus guaranteeing clear distinguishable pathways.

Let Ab(t) (aB(t)) denote the random variable, that there is an AB mutant until time t via pathway Ab (aB). Thus, $\neg$Ab(t) corresponds to the random variable, that there is no AB mutant until time t vial pathway Ab. Then the probability, that the first mutant arises exactly at time t via pathway Ab (i.e. not via pathway aB beforehand) is

$$\begin{aligned}\rho_{Ab}(t) =& P(Ab(t) \cap \neg Ab(t-1) \cap \neg aB(t-1)) \\ =& P(\neg Ab(t-1) \cap \neg aB(t-1)) - P(\neg Ab(t) \cap \neg aB(t-1)). \end{aligned} \tag{A.6}$$

The first term is calculated by the pgf as in Eq. (A.1). For the second term however, the time points for the different pathways are different. Let us derive a recursive function for this second term at this point. To do so, let us first consider the extinction probability for the subprocess of $Ab \to AB$, where the process starts with one Ab individual. As discussed previously, this extinction probability within $t-1$ time steps can be recursively calculated by its probability generating function

$$f_{Ab}^{\circ(t-1)} = d_{Ab} + (1 - b_{Ab} - d_{Ab}) f_{Ab}^{\circ(t-2)} + b_{Ab} \left((1-\mu_B^A) f_{Ab}^{\circ(t-2)} + \mu_B^A e_{AB} \right)^2, \tag{A.7}$$

with $f_{Ab}^{\circ(0)} = 1$. Similarly, the extinction probability for the subprocess $aB \to AB$ within $t-2$ time steps can be calculated recursively using the probability generating function for aB

$$f_{aB}^{\circ(t-2)} = d_{aB} + (1 - b_{aB} - d_{aB}) f_{aB}^{\circ(t-3)} + b_{aB} \left((1-\mu_A^B) f_{aB}^{\circ(t-3)} + \mu_A^B e_{AB} \right)^2, \tag{A.8}$$

with $f_{aB}^{\circ(0)} = 1$. When we now consider the extinction probability of the whole process starting with an individual of type ab, we see that it can either go extinct right away, or if it divides we can refer to the individual extinction probabilities for the different types (in case of mutation), i.e. their probability generating functions

$$\begin{aligned}\bar{f}_{ab}^{\circ(t)} :=& d_{ab} + (1 - b_{ab} - d_{ab}) \bar{f}_{ab}^{\circ(t-1)} + b_{ab} \left((1 - \mu_A - \mu_B) f_{ab}^{\circ(t-1)} + \mu_A f_{Ab}^{\circ(t-1)} + \mu_B f_{aB}^{\circ(t-2)} \right)^2 \\ =& \bar{f}_{ab}(\bar{f}_{ab}^{\circ(t-1)}, f_{Ab}^{\circ(t-1)}, f_{aB}^{\circ(t-2)}), \end{aligned} \tag{A.9}$$

with $\bar{f}^{\circ(0)}_{ab} = 1$, $f^{\circ(0)}_{Ab} = 1$, and $f^{\circ(0)}_{aB} = 1$. Note, that in contrast to the normal probability generating function, here the probability generating function for type aB has one time step less, which agrees with the second term in A.6. To not confuse this modified probability generating function with the common one, we use the *bar-notation*. Again, no probability generating function for the AB-type is necessary, since the actual extinction probability for this type is used.

We define this recursive function as

$$\bar{f}^{\circ(t)}_{ab}(s_{ab}, s_{Ab}, s_{aB}, s_{AB}) := \bar{f}^{(Ab)}(t). \tag{A.10}$$

The index Ab denotes, that this is the modified probability generating function for the pathway via Ab.

With this we now describe the algorithm for the path probability.

1. Calculate the extinction probability of the final mutant type AB as above.

2. Until some t_{max} calculate recursively $f(t)$ as explained above in Eq. A.4.

3. Until some t_{max} calculate recursively

$$\begin{aligned} f^{\circ(t)}_{aB} &= d_{aB} + (1 - b_{aB} - d_{aB}) f^{\circ(t-1)}_{aB} + b_{aB}\left((1-\mu^B_A) f^{\circ(t-1)}_{aB} + \mu^B_A e_{AB}\right)^2, \\ f^{\circ(t)}_{Ab} &= d_{Ab} + (1 - b_{Ab} - d_{Ab}) f^{\circ(t-1)}_{Ab} + b_{Ab}\left((1-\mu^A_B) f^{\circ(t-1)}_{Ab} + \mu^A_B e_{AB}\right)^2, \end{aligned} \tag{A.11}$$

$$\begin{aligned} \bar{f}^{(Ab)}(t) := \bar{f}^{\circ(t)}_{ab} &= d_{ab} + (1 - b_{ab} - d_{ab}) \bar{f}^{\circ(t-1)}_{ab} \\ &+ b_{ab}\left((1 - \mu_A - \mu_B)\bar{f}^{\circ(t-1)}_{ab} + \mu_A f^{\circ(t-1)}_{Ab} + \mu_B f^{\circ(t-2)}_{aB}\right)^2 \end{aligned}$$

   where $f^0_{aB} = f^{-1}_{aB} = f^0_{Ab} = f^0_{ab} = 1$. Note, that the only difference is that the probability generating function of types not along the pathway considered is one time step behind (marked in red). This is also the reason, why there are two initial conditions needed for type aB.

4. The probability to get the final, successful AB mutant exactly at time t via path Ab and not getting a successful AB mutant beforehand is then computed by

$$\rho_{Ab} = f^N(t-1) - \left(\bar{f}^{(Ab)}(t)\right)^N. \tag{A.12}$$

Analogously one can calculate the path probability for reaching the final mutant via aB. Note, that while this computation gives the correct path probabilities, the sum over all paths

can be slightly greater than the overall time distribution. This is due to the fact, that in time discrete systems the final mutant can be reached by different pathways at the same time. In the description here, such cases count for all pathways that succeed at the time.

References

[1] K. B. Athreya and P. E. Ney. *Branching Processes*. Springer, Berlin, 1972.
